# Bats as Reservoir Hosts of Human Bacterial Pathogen, *Bartonella mayotimonensis*

**DOI:** 10.3201/eid2006.130956

**Published:** 2014-06

**Authors:** Ville Veikkolainen, Eero J. Vesterinen, Thomas M. Lilley, Arto T. Pulliainen

**Affiliations:** University of Turku, Turku, Finland (V. Veikkolainen, E.J. Vesterinen, T.M. Lilley, A.T. Pulliainen);; University of Helsinki, Helsinki, Finland (A.T. Pulliainen)

**Keywords:** bats, bacteria, metagenomics, endocarditis, *Bartonella*, Northern Hemisphere

## Abstract

A plethora of pathogenic viruses colonize bats. However, bat bacterial flora and its zoonotic threat remain ill defined. In a study initially conducted as a quantitative metagenomic analysis of the fecal bacterial flora of the Daubenton’s bat in Finland, we unexpectedly detected DNA of several hemotrophic and ectoparasite-transmitted bacterial genera, including *Bartonella*. *Bartonella* spp. also were either detected or isolated from the peripheral blood of Daubenton's, northern, and whiskered bats and were detected in the ectoparasites of Daubenton's, northern, and Brandt's bats. The blood isolates belong to the *Candidatus*-status species *B. mayotimonensis*, a recently identified etiologic agent of endocarditis in humans, and a new *Bartonella* species (B. naantaliensis sp. nov.). Phylogenetic analysis of bat-colonizing *Bartonella* spp. throughout the world demonstrates a distinct *B. mayotimonensis* cluster in the Northern Hemisphere. The findings of this field study highlight bats as potent reservoirs of human bacterial pathogens.

## Abstract 2

Bats can be potent reservoirs of human bacterial pathogens.

The 1,100 species of bats (*1*) constitute ≈20% of known mammalian species and are outnumbered only by animals in the order Rodentia. Bats play a vital role in natural ecosystems in arthropod suppression, seed dispersal, and pollination. Modern-day economies also benefit from these voracious predators of crop and forest pests (*2*). However, bats have been implicated as reservoir hosts for viral human pathogens, such as paramyxoviruses (*3*) and rabies virus and related lyssaviruses (*4*). Compelling evidence also indicates that bats carry asymptomatically some of the most deadly viruses, including Marburg (*5*) and Ebola (*6*) viruses. Whether bats carry clinically significant bacterial pathogens is unknown.

The development of next-generation sequencing techniques has revolutionized biological science. It is now possible—and cost-friendly—to gain access to massive amounts of qualitative and quantitative sequencing data in a short time without a priori knowledge of the sequence (*7*). Most bacteria do not grow on laboratory media, and next-generation sequencing technologies have proven useful for studying bacterial species diversity and dynamics, even in complex systems like the gut (*8*). Our initial objective in 2010 and 2011 was to conduct a quantitative metagenomic analysis of the fecal bacterial flora of the Daubenton’s bat (*Myotis daubentonii*) in Finland. Unexpectedly, we found that the fecal material contained DNA of several hemothrophic and ectoparasite-transmitted bacterial genera, such as *Bartonella*. This DNA may originate either from bleeding into the intestine or from the insect prey of the bats that includes the abundant bloodfeeding bat ectoparasites. Therefore, the study further focused on detecting and isolating *Bartonella* spp. from peripheral blood and ectoparasites of several bat species in Finland in 2012.

## Materials and Methods

*Bartonella* spp. nucleotide sequences have been deposited in GenBank under accession nos. KF003115–KF003145. The metagenomic reads are stored at the National Center for Biotechnology Information Sequence Read Archive under BioProject SRP023235 (accession nos. experiment: SRX286839, run: SRR868695). We have described the detailed protocols, including bat sampling for peripheral blood, fecal droppings, and ectoparasites; metagenomic analysis of fecal DNA; isolation of *Bartonella* from peripheral blood; extraction of DNA from bat blood, ectoparasites, and *Bartonella* isolates; *Bartonella* and ectoparasite PCR analyses; transmission electron microscopy; and nucleotide sequence and phylogenetic analyses in the Technical Appendix.

## Results

### Quantitative Metagenomic Analysis of DNA from Bat Feces

**Figure 1:**
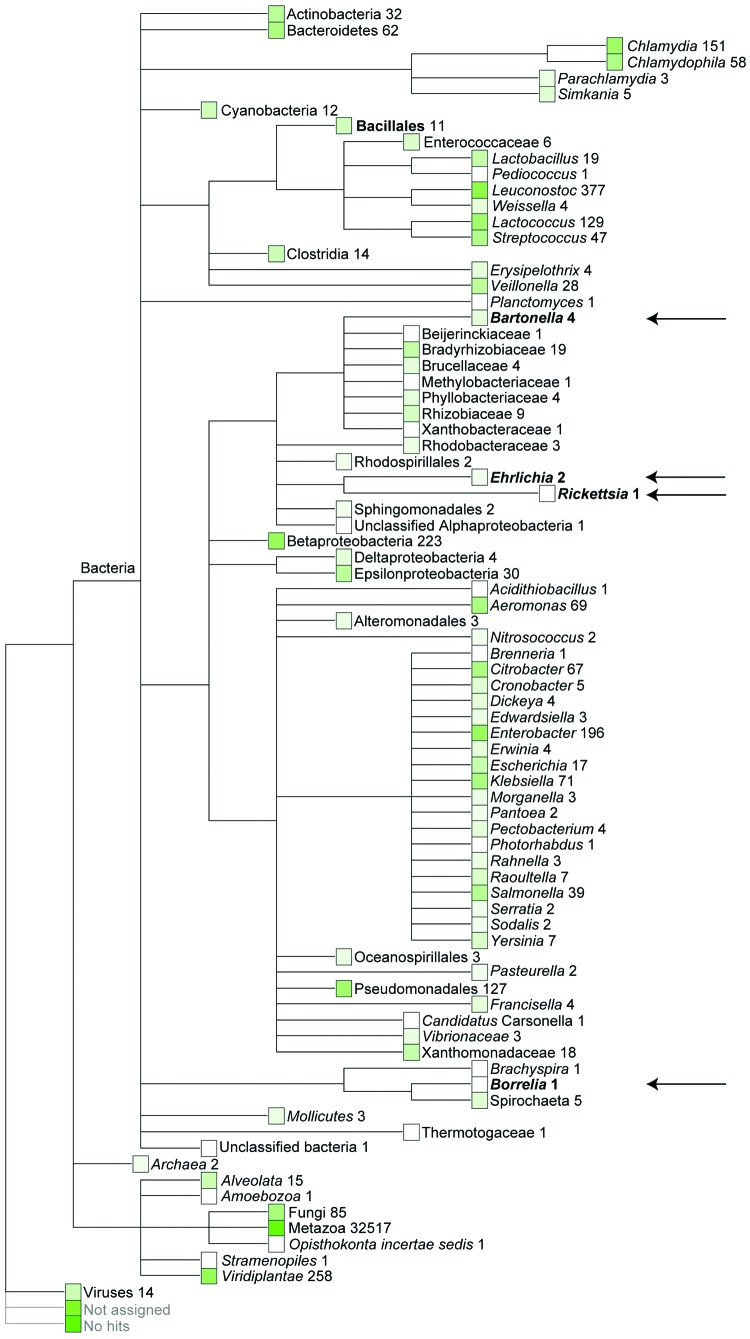
Quantitative metagenomic analysis of the fecal DNA of the Daubenton’s bat. The sequences (>50 bp) were assigned on the basis of best E-value BLASTN scores (http://blast.ncbi.nlm.nih.gov/blast.cgi) in GenBank. Numbers refer to the amount of sequences assigned to a given taxon. No hits refers to sequences that had no similarity to any sequences in GenBank. Not assigned refers to sequences that had similarity in GenBank but they could not be reliably assigned to any organism. Arrows mark the ectoparasite-transmitted bacterial genera, which unexpectedly were detected in the bat fecal DNA preparation.

We obtained ≈200,000 high-quality sequences (average length 167 bp) from DNA sequencing of fecal material from a Daubenton’s bat (Technical Appendix Figure 1). Sequences (>50 bp) were assigned on the basis of best E-value BLASTN scores (http://blast.ncbi.nlm.nih.gov/blast.cgi) in GenBank. The most abundant non-metazoan sequence matches were with bacteria. The genera *Leuconostoc*, *Enterobacter*, *Lactococcus*, and *Chlamydia* dominated (Figure 1). Surprisingly, the fecal material also contained DNA of the ectoparasite-transmitted genera, such as the hemotrophic bartonellae (*9*). It was thought that this DNA originated either from bleeding into the intestine or from the insect prey of the bats that includes the abundant bloodfeeding bat ectoparasites. PCR verified the presence of *Bartonella* DNA in the bat fecal material. The transfer messenger RNA gene (*ssrA*) (*10*) could be amplified and was sequenced from the fecal material of 1 Daubenton’s bat, 1 northern bat (*Eptesicus nilssonii*), and 1 Brandt’s bat (*Myotis brandtii*) (no. 2771, no. 2788, and no. 2786, respectively; Technical Appendix Table 1). The obtained 218-bp *ssrA* sequences were 100% identical. The closest matches in GenBank, with a similarity score of 94.8% (183/193 bp), were *B. tamiae* Th339 (GenBank accession no. JN029780) and Th307 strains (GenBank accession no. JN029778) isolated from 2 humans in Thailand (*11*).

### *Candidatus* Status Species B. mayotimonensis and Novel *Bartonella* Species

Bats belonging to the 4 most prevalent bat species in Finland were captured in August and September 2012 at 3 locations in southwestern Finland (Technical Appendix Table 1). Culturing of peripheral blood samples of 5 Daubenton’s bats and 1 northern bat yielded distinct colonies. The isolates were identified as *Bartonella* spp. by sequencing a PCR-amplified 485-bp fragment containing the hypervariable regions V6–V8 of the 16S rRNA gene. Overall health of the bats as analyzed by body condition indexing was not affected by the *Bartonella* infection (Technical Appendix Table 1).

The 16S rRNA gene sequences are highly conserved within the genus *Bartonella* and thus not robust in differentiating species (*12*). Therefore, we sequenced PCR-amplified fragments of the RNA polymerase β-subunit gene (*rpoB*), citrate synthase gene (*gltA*), filamenting temperature-sensitive mutant Z gene (*ftsZ*), VirB type IV secretion system VirB4 component gene (*virB4*), hypervariable region 2 of the 16S-23S rRNA intergenic spacer region (ISR), and *ssrA* (Technical Appendix Table 2). Sequencing of *rpoB* was first conducted on all 28 clonal isolates. Three distinct *rpoB* alleles were identified (Technical Appendix Table 1). The multilocus sequence analysis (MLSA) was completed on 1 *rpoB*-1 allele isolate (clone 3, bat no. 1157, referred to hereafter as 1157/3), 1 *rpoB*-2 allele isolate (clone 1, bat no. 2574, referred to hereafter as 2574/1), and 1 *rpoB*-3 allele isolate (clone 1, bat no. 1160, referred to hereafter as 1160/1). Thin-section transmission electron micrographs of these isolates are shown in the Technical Appendix Figure 2. No major pili or fimbriae-like structures were detected on the surface of the rod-shaped bacteria.

Results of BLASTN homology searches performed in January 2013 are shown in Technical Appendix Table 3. ISR is a robust species discriminatory marker within the genus *Bartonella* (*13*,*14*). ISR of the strain 2574/1 did not have any hits, whereas ISR of strains 1157/3 and 1160/1 had on1y 1 hit in GenBank *Candidatus* B. mayotimonensis (*15*), with high sequence similarity scores. Sequence analyses of the other MLSA markers (Technical Appendix Table 3) further indicate that isolates 1157/3 and 1160/1 belong to the *Candidatus*-status species B. mayotimonensis and that strain 2574/1 belongs to a new *Bartonella* species. Indeed, the lowest pairwise genetic distance values with the concatenated *rpoB*, *gltA*, 16S rRNA, and *ftsZ* sequence fragments of the bat strains 1157/3 and 1160/1 in the genus *Bartonella* were 0.040 and 0.038, respectively, with *Candidatus* B. mayotimonensis (Technical Appendix Table 4). Because the distance value 0.05 is the recommended cutoff value for species delineation (*16*), the bat isolates 1157/3 and 1160/1 classify as strains of the *Candidatus*-status species B. mayotimonensis. The bat strain 2574/1 belongs to a new *Bartonella* species because the lowest genetic distance value in the genus *Bartonella* was 0.070 with *B. washoensis*, above the 0.05 cutoff value (*16*).

**Figure 2:**
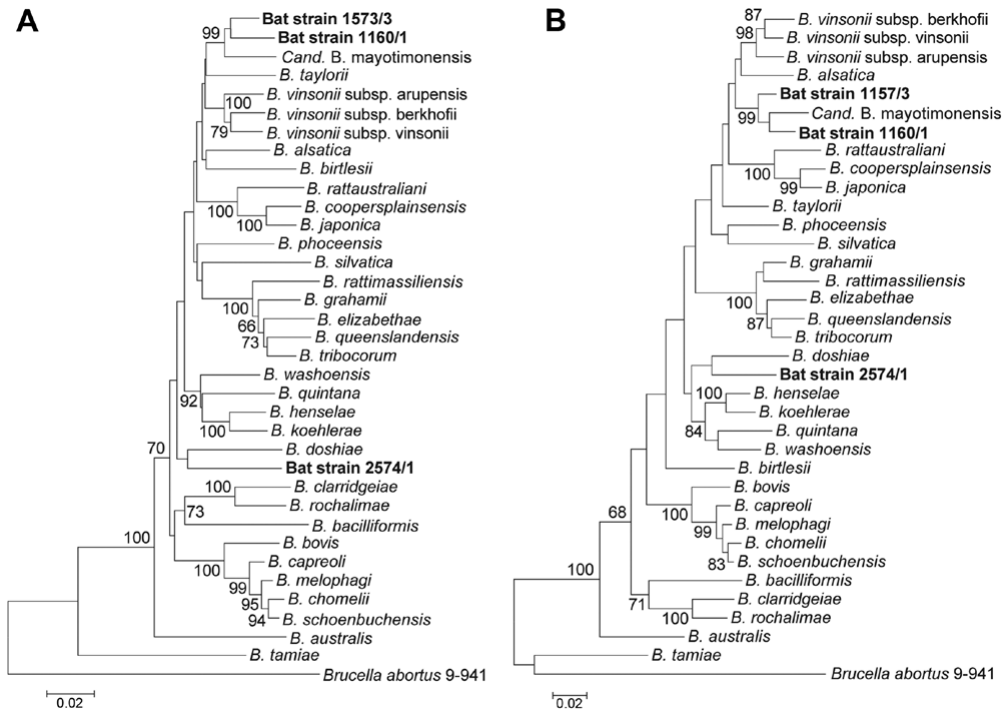
Phylogenetic positions of the bat blood isolates among members of the genus *Bartonella*. Neighbor-joining (A) and maximum-likelihood (B) trees are based on the alignment of concatenated sequences of 4 multilocus sequence analysis markers (*rpoB*, *gltA*, 16S rRNA, and *ftsZ*). Sequence information from the type strains of all known *Bartonella* species and from the *Candidatus* B. mayotimonensis human strain was included into the analysis (Technical Appendix Table 5). Numbers on branches indicate bootstrap support values derived from 1,000 tree replicas. Bootstrap values >60 are shown. Scale bar indicates nucleotide substitutions per site.

Figure 2 shows the phylogenetic position of the bat *Bartonella* isolates based on comparisons of concatenated sequences of *rpoB*, *gltA*, 16S rRNA and *ftsZ*, available for *Candidatus* B. mayotimonensis (*15*) and all type strains of the *Bartonella* species (Technical Appendix Table 5). The neighbor-joining and maximum-likelihood trees demonstrate that bat isolates 1157/3 and 1160/1 cluster with *Candidatus* B. mayotimonensis with high bootstrap values in a distinct phylogenetic position. The new *Bartonella* species (strain 2754/1) clearly diverges from the other bat isolates.

### Bat Ectoparasite Flies and Fleas as Vectors for Transmitting *Bartonella*

We sequenced a PCR-amplified fragment of the mitochondrial cytochrome c oxidase subunit I (*17*) and also used visual inspection to identify the ectoparasites of 18 bats (o Technical Appendix Table 1). Ectoparasite DNA preparations of 2 fleas and 10 flies were analyzed with a PCR protocol targeting the *Bartonella rpoB*. The blood isolate *rpoB* alleles 1 and 2 were detected in samples from 1 flea and 2 flies, respectively (Technical Appendix Table 1). In addition, 2 novel *rpoB* alleles were detected. The *rpoB*-5 allele detected in a fly sample is distantly related to the currently known *Bartonella rpoB* sequences. The highest BLASTN sequence identity score with the *rpoB*-4 allele detected in a flea sample, and from 1 blood DNA preparation of a culture-negative whiskered bat (no. 1156, Technical Appendix Table 1), was 97.8% (397/406 bp) with the corresponding fragment (FJ376736) of *Candidatus* B. mayotimonensis. This is a higher value than with the *rpoB*-1 and *rpoB*-2 alleles. Moreover, a partial 338-bp *gltA* fragment could be amplified from the *rpoB*-4–positive flea sample. The highest BLASTN sequence identity score with *Candidatus* B. mayotimonensis was 93.2% (315/338 bp), which is higher than with the isolates 1157/3 (92.0%, 311/338 bp) and 1160/1 (92.3%, 312/338 bp). The data further support the conclusion that bats are reservoir hosts of *B. mayotimonensis* and indicate that the bat flies and fleas transmit *Bartonella* spp. to new hosts.

### Phylogenetic Analysis of *Bartonella* spp. that Colonize Bats Worldwide 

**Figure 3:**
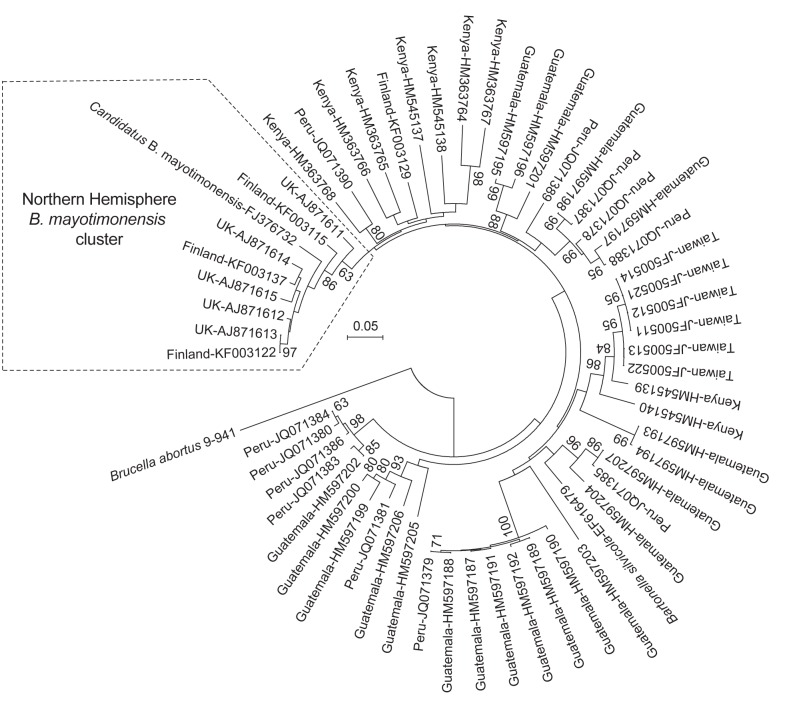
Phylogenetic analysis of bat-colonizing *Bartonella* spp. found worldwide demonstrates a distinct *B. mayotimonensis* cluster in the Northern Hemisphere. Maximum composite likelihood–based neighbor-joining tree is based on the alignment of the *gltA* multilocus sequence analysis marker. Information from *Bartonella gltA* sequences from bat blood isolates or from minced tissues of bats or from bat ectoparasites was included in the analysis. GenBank accession numbers of the sequences are shown after the country of origin. Numbers on branches indicate bootstrap support values derived from 1,000 tree replicas. Bootstrap values >60 are shown. Scale bar indicates nucleotide substitutions per site.

A maximum composite likelihood–based neighbor-joining tree (Figure 3) was constructed on the basis of 253-bp *gltA* sequences obtained from *Bartonella* that infect bats in the United Kingdom (*18*), Kenya (*19*), Guatemala (*20*), Taiwan (*21*), and Peru (*22*). The 5 *Bartonella*-like bacteria detected in minced heart tissues in the United Kingdom (*18*), the *B. mayotimonensis* isolates from Finland, and the strain detected in 1 bat flea in Finland clustered in a distinct phylogenetic position away from the bat isolates and strains of the Southern Hemisphere. The new *Bartonella* species (strain 2574/1) does not belong to the Northern Hemisphere *B. mayotimonensis* cluster. Remarkably, the 253-bp *gltA* fragment of 1 of the *Bartonella*-like bacteria detected in minced heart tissue of a common noctule (*Nyctalus noctule*) (Cornwall-M451, AJ871615) yielded a 95.3% (241/253 bp) sequence identity score, compared with the corresponding fragment (FJ376732) of *Candidatus* B. mayotimonensis. This value is significantly higher than those obtained with corresponding *gltA* fragments of Finland bat isolates 1157/3 (92.9%, 235/253) or 1160/1 (94.1%, 238/253). The UK *gltA* data further support the conclusion that bats are reservoir hosts of *B. mayotimonensis*. Most importantly, bats appear to be reservoir hosts of *B. mayotimonensis* only in the Northern Hemisphere.

*Bartonella naantaliensis* (naan.tali´en.sis. N.L. fem. adj. n. *naantaliensis* of or belonging to Naantali) is the name proposed to highlight the municipality where the bat was trapped from which the type strain was isolated. The type strain is 2574/1. Its partial 16S rRNA gene nucleotide sequence is deposited in GenBank (accession no. KF003116).

## Discussion

*Bartonella* spp. are facultative intracellular bacteria that typically cause long-lasting hemotrophic bacteremia in their mammalian reservoir hosts, such as rodents (*9*). The relapsing bacteremia can last weeks, months, or even years, thereby favoring transmission by bloodfeeding arthropods. In recent years, increasing numbers of *Bartonella* spp. have been implicated as zoonotic human pathogens. A frequent symptom is endocarditis, usually suspected in cases in which conventional culture-based diagnostics fail. The most prevalent endocarditis-causing species are *B. quintana* (*23*,*24*) and *B. henselae* (*25*), but *B. elizabethae* (*26*), *B. alsatica* (*27*), *B. koehlerae* (*28*), *B. vinsonii* subsp. *berkhoffii* (*29*), and *B. vinsonii* subsp. *arupensis* (*30*) also have been detected or isolated. Recently, a new type of *Bartonella* was detected in a resected aortic valve tissue of a human endocarditis patient (*15*). A species name, *Candidatus* B. mayotimonensis was proposed because a pure microbiological culture was not obtained. The reservoir host in nature also remained elusive. As part of a study designed to characterize the microbiome of bats, bacteria that belong to the *Candidatus*-status species B. mayotimonensis were either detected or isolated from peripheral blood samples and the ectoparasites of bats. In addition, a new *Bartonella* species (strain 2574/1) was isolated from the blood and detected from the ectoparasites.

The ad hoc committee to reevaluate the species definition in bacteriology has proposed that descriptions of novel species could be based solely on gene sequence analyses (*31*). In the current study, 6 genes, including the robust *Bartonella* spp. discriminatory marker, the ISR, were used (*13*,*14*). It is remarkable that ISR of the 2574/1 isolate did not have any hits, whereas ISRs of 1157/3 and 1160/1 isolates had only 1 hit in GenBank, *Candidatus* B. mayotimonensis. If *gltA* shares <96.0% and *rpoB* <95.4% nt sequence similarity with those of the validated species, the newly encountered *Bartonella* strain can be considered a new species (*32*). According to these criteria, which were proposed in 2003 when half of the currently known species were known, the bat isolate 2574/1 is a new *Bartonella* species. The bat isolates 1157/3 and 1160/1 belong to the *Candidatus*-status species B. mayotimonensis on the basis of the *rpoB* sequences but would belong to a new *Bartonella* species on the basis of the *gltA* sequences. Because the species classification gave contradictory results, sequence analyses of other MLSA markers and phylogenetic analyses were performed. In addition, we used 4 concatenated MLSA markers to determine pairwise genetic distance values to the known members of the genus. The bat isolate 1157/3 and 1160/1 *ftsZ* sequences had a significantly higher sequence similarity with *ftsZ* of *Candidatus* B. mayotimonesis than with any other type strain sequence. The neighbor-joining and maximum-likelihood phylogenetic trees with the concatenated *rpoB*, *gltA*, 16S rRNA and *ftsZ* sequences both demonstrated that the bat isolates 1157/3 and 1160/1 cluster with *Candidatus* B. mayotimonensis with high bootstrap values in a distinct phylogenetic position. Moreover, the genetic distance values demonstrate that the bat isolates 1157/3 and 1160/1 classify as strains of the *Candidatus*-status species B. mayotimonensis. We propose that the bat isolate 1160/1 is the type strain of *B. mayotimonensis*.

Findings of the study raised an interesting question: how could *Bartonella* spp., or any other hemotrophic bacterium, be transmitted from the bat into the human host? Daubenton’s bats prefer to roost in abandoned woodpecker cavities and bird boxes, whereas the other bat species are often found in the attics of houses in close proximity to humans. Given that *Bartonella* spp. are hemotrophic, transmission through bat bite and saliva is not considered likely. Moreover, at Turku University Central Hospital, which is responsible for a population base of 500,000, only 2 or 3 patients per year are admitted with a bat bite (J. Oksi, pers. comm.). These numbers probably reflect the frequency of bat bites in most countries of the Northern Hemisphere. We propose that fecal droppings of blood-fed bat ectoparasites might transmit *Bartonella* spp. into the human host, assisted by superficial scratching or tissue trauma of the skin. The presence of viable bacteria in feces of body lice (*Pediculus humanus*) that have been feeding on *B. quintana*–infected rabbits is well documented (*33*,*34*). Similar observations have been reported for the feces of experimentally infected cat fleas (*Ctenocephalides felis*) (*35*,*36*). Most importantly, intradermal injection of feces from fleas that had fed on a *B. henselae*–infected cat led to bacteremia in a pathogen-free cat (*37*). Ectoparasite bite–mediated transmission is also possible, but the bat bugs (*Cimex* spp.) known to also feed on humans were not analyzed in the current study.

The reported metagenomic analysis of bat fecal material indicates that bats are reservoir hosts for several pathogenic bacterial genera. No comprehensive study has been published on the bacterial flora of bats in light of its zoonotic threat to humans. The major research focus has been on viruses, and several deadly viruses have been detected or isolated (*3*–*6*). One of the main conclusions from these studies is that bats tolerate their deadly companions relatively well, a feature that has been discussed in the context of long evolutionary history of bats (*38*). Bats are also highly mobile and long-lived, ideal as pathogen reservoirs. Metagenomics-driven approaches should be continued to assess the pathogenic potential of bacteria that colonize bats.

Technical Appendix Materials and methods, Technical Appendix Tables 1–5 and Technical Appendix Figures 1 and 2.
