# Supplementary material for: Bats as Reservoir Hosts of Human Bacterial Pathogen, Bartonella mayotimonensis
Source: Emerg Infect Dis. 2014 Jun;20(6):960–7. doi: 10.3201/eid2006.130956 (PMC4036794; doi:10.3201/eid2006.130956)
Supplement: Technical Appendix — Materials and methods, Technical Appendix Tables 1–5 and Technical Appendix Figures 1 and 2. [file 13-0956-Techapp-s1.pdf]

Article DOI: http://dx.doi.org/10.3201/eid2006.130956

Bats as Reservoir Hosts of Hhuman Bacterial Pathogen, *Bartonella mayotimonensis*

Technical Appendix

Bat Sampling for Peripheral Blood, Fecal Droppings, and Ectoparasites

Bats were caught with a combination of mist nets and harp trap (Animal Ethics Committee license no. ESLH-YM-2007-01055). Two mist nets were positioned on each side of the harp trap. A Sussex Autobat siren (*1*), which produces species-specific ultrasound social calls, was placed in the center of the harp trap to attract the bats. This multitrap combination was placed across the flying corridor of bats commuting between roosts and foraging areas. Caught bats were visually identified to species, banded, and measured for mass and forearm length. The tail skin membrane was wiped with cotton sticks soaked in 75% (v/v) ethanol. The blood sample was collected into a 75-µL heparinized capillary tube from the interfemoral vein after lancing with a 25-gauge needle. Blood samples were stored on ice until culturing. Fur ectoparasites collected from bats were surface sterilized for 15 min in 75% (v/v) ethanol followed by a wash with phosphate-buffered saline (PBS). The ectoparasites were stored dry at –80°C until isolation of DNA. Fecal droppings were collected from holding bags where the bats were kept during the capture period or straight from the bats during handling. All bats were released after sampling.

Metagenomic Analysis of Fecal DNA

Fecal samples were processed in the Herbarium laboratory at the University of Turku (Turku, Finland), where so far only plant specimens have been handled. Fecal DNA was extracted using QIAamp DNA Stool Mini Kit (Qiagen Inc., Valencia, CA, USA, catalog no.51504). Negative control extraction containing all the chemicals but no fecal pellet was performed alongside to monitor for contamination of the extraction chemicals. The DNA fragmentation and library preparation was performed in the TegLab facilities (Laboratory of Genetics,

University of Turku) following Ion Torrent user guide (publication part no. 4471989 rev. B). Negative control reaction was performed to monitor for contamination of the chemicals used. Adapter ligation success was visually inspected under UV light using a 2% (w/v) agarose gel stained with 0.5 μg/mL (w/v) of ethidium bromide. The DNA library was amplified with the following setup: 5 μL library was added to a master mix consisting of 5 U of Herculase II polymerase (Agilent Technologies, Santa Clara, CA, USA, catalog no. 600677), 1× Herculase II reaction buffer, 25 mM each dNTP, 10 μM each primer, and added PCR-grade water up to 50 μL. Amplification step generated millions of DNA copies, which include the binding sites necessary for subsequent Ion Torrent sequencing. The thermocycling profile included a 30 s denaturation at 98°C followed by 15 cycles consisting of a 20 s denaturation at 98°C, a 30 s annealing at 64°C, and a 30 s elongation at 72°C. Final elongation was conducted at 72°C for 5 min. To clean the amplified library of leftover adapters and primer-dimers, size-selection was done by separating the entire library using 2% (w/v) Size-Select Agarose E-Gel and E-Gel Electrophoresis System (Life Technologies, Carlsbad, CA, USA, catalog nos. G6610-02 and G6500) following the manufacturer's instructions. The library pool stock was then diluted to a final concentration of 26 pM. For template preparation, an 18-μL aliquot of the library dilution ($\approx 2.8 \times 10^8$ molecules) was transferred into the sequencing reaction setup. Emulsion PCR and Ion Torrent Sequencing was carried out on a 314 chip according to the manufacturer's protocol (publication part no. 4471974 rev. C). Performance of the Ion Torrent Personal Genome Machine is shown in Technical Appendix Figure 1. The resulting reads were trimmed of sequencing adapters and poor-quality parts by using 0.05 error probability limit and then the reads <50 bp were excluded by using the software Geneious Pro (Geneious version 6.1, Biomatters) available at www.geneious.com/. Subsequent analyses were carried out by using super computer clusters at the IT Center for Science (Espoo, Finland, www.csc.fi) and at Finnish Grid Infrastructure (www.csc.fi/english/collaboration/projects/fgi). Sequences were assigned to GenBank reference database sequences using the BLASTN 2.2.25+ algorithm. MetaGenome Analyzer software (MEGAN v4.70.4) available at http://ab.inf.uni-tuebingen.de/software/megan/ was used to visualize the results.

Isolation of *Bartonella* from Peripheral Blood

Blood samples were cultured within 3–6 hours after blood sampling. Blood-filled heparinized capillary tubes were emptied into 500 μL of PBS on ice. Broad-spectrum antifungal

compound amphotericin B (Fungizone; Sigma, catalog no. A2942) was added at a concentration of 10 μg/mL (w/v). 400 μL aliquots of the blood samples were cultured on Columbia Blood Agar Base (CBA) (Difco, catalog no. 279240) supplemented with 5% (v/v) of defibrinated sheep blood. The remaining samples were stored at –80°C for DNA isolation. The plates were incubated in a humified 5% CO_2 atmosphere at 37°C up to 1 month. Individual colonies from the primary plates (passage 0) were subcultured on fresh CBA blood plates. After 1 week of incubation as described above, the clonal isolates were suspended in 1 mL of Todd Hewitt Broth (Beckton Dickinson , Franklin Lakes, NJ, USA, catalog no. 249210) supplemented with 0.5% (w/v) yeast extract (Biokar Diagnostics, Beauvais, France, catalog no. A1202HA) [THY] and 25% (v/v) of glycerol. These solutions were stored at –80°C as passage 1 stocks.

Extraction of DNA from Bat Ectoparasites, Blood, and *Bartonella* Isolates

Ectoparasites were mechanically disrupted with Kimble Kontes pellet pestle (Sigma) in 200 μL PBS. One hundred microliters of bat blood–PBS solution (see above) was diluted with 100 μL PBS. First, the samples were incubated for 10 min at room temperature in 2% (w/v) sodium dodecyl sulphate, and then, after 3 U Proteinase K (Finnzymes) was added, in a shaker at 60°C for 2 h. After incubation, 150 μL of saturated NaCl (6 M) was added, the samples were vortexed for 30 sec and centrifugated at 16100 rcf for 30 min. From the supernatant, the DNA was precipitated with 200 μL of isopropanol overnight at –20°C. The next day, the precipitated DNA was pelleted with centrifugation at 16100 rcf and washed with 200 μL of ice cold 70% (v/v) ethanol. The DNA pellets were air-dried and dissolved in sterile water. Passage 2 clonal isolates were harvested from 5-day-old CBA blood plates into sterile PBS. Bacteria were pelleted by centrifugation (16100 rcf, 2 min). Bacterial pellets were resuspended in 1 mL of 25 mM Tris-HCl, 50 mM glucose, 10 mM EDTA (pH 8.0) containing 500,000 U of lysozyme and 100 U of RNAse A. The suspensions were incubated at 37°C for 2 h. Sodium dodecyl sulphate was added to 1.0% (w/v), and the proteins were removed by 2 phenol and subsequent 2 chloroform precipitations. 0.11 volume of 3 M NaOAc (pH 5.2) was added. The DNA was precipitated, washed and dissolved as above, except 2.2 volumes of ice-cold 99% (v/v) ethanol was added to precipitate the DNA.

Bartonella and Ectoparasite PCR Analyses

The PCR reactions were carried out in a total volume of 50 mL, containing 2 mM primers (Technical Appendix Table 2), 50 mM of each dNTP, 1 U of DyNAzyme II DNA Polymerase (Thermo Scientific), and 100–250 ng of template DNA or water (negative control). DNA from *Bartonella henselae* Houston-1 was used as a positive *Bartonella* control. All of the PCRs were run under the same conditions with an initial denaturation at 95°C for 1 min, followed by denaturation at 95°C for 30 s, annealing at 55°C for 15 s, and extension at 72°C for 1 min. Amplification was completed by 39 additional cycles at 72°C for 1 min and final extension at 72°C for 10 min.

Transmission Electron Microscopy

Bacteria were harvested from 5-day-old CBA blood plates into sterile PBS. Bacteria were pelleted by centrifugation (16100 rcf, 2 min) and fixed with 5% (v/v) glutaraldehyde in 0.16 M s-collidine buffer pH 7.4. Bacterial pellets were embedded in epoxy resin, and the blocks were cut by using an ultra microtome (Leica Ultracut UCT). 70-nm ultrathin sections were mounted on formvar-coated copper grids. The ultrathin sections were stained with 1% (w/v) uranyl acetate for 30 min at 20°C and 0.3% (w/v) lead citrate for 3 min at 20°C. The grids were examined using electron microscopes JEM-1200EX and JEM-1400 Plus, JEOL, Tokio, Japan.

Nucleotide Sequence and Phylogenetic Analyses

To incorporate all *Bartonella* species and Candidatus *B. mayotimonensis* into the type strain phylogeny (Figure 2) and the pairwise genetic distance value calculations (Technical Appendix Table 4), *rpoB* sequences were trimmed to 406-bp fragments (corresponds to nucleotide positions 246–651 of *B. alsatica rpoB*, AF165987), *gltA* sequences down to 311–312-bp fragments (corresponds to nucleotide positions 4–315 of *B. alsatica gltA*, AF204273), 16S rRNA sequences down to 483–85-bp fragments (corresponds to nucleotide positions 881–1365 of *B. alsatica rpoB*, AJ002139), and *ftsZ* sequences down to 280-bp fragments (corresponds to nucleotide positions 61–340 of *B. alsatica ftsZ*, AF467763). GenBank accession numbers of the type strain sequences are shown in Technical Appendix Table 5. Phylogenetic analysis of the worldwide bat-colonizing *Bartonella* strains (Figure 3) was performed by using the *gltA* sequences trimmed down to 253-bp fragments (corresponds to

nucleotide positions 4–256 of *B. alsatica gltA*, AF204273). Phylogenetic analyses were performed by using Molecular Evolutionary Genetics Analysis (MEGA) 5.2.1 (www.megasoftware.net/). To this end, the sequences were first aligned with ClustalW. The neighbor-joining trees were constructed by using the maximum composite likelihood method with 1,000 replicas. The maximum-likelihood trees were constructed using the Tamura-Nei method with 1,000 replicas and nearest-neighbor-interchange as the maximum-likelihood heuristic method with the default option to construct the initial tree.

Reference

1. Hill D, Greenaway F. Effectiveness of an acoustic lure for surveying bats in British woodlands. Mammal Rev. 2005;35:116–22. http://dx.doi.org/10.1111/j.1365-2907.2005.00058.x

Technical Appendix Table 1. Bat sampling and PCR-detection and isolation of *Bartonella* spp.*

Sample type, bat species	Capture date	Capture location	Band no., sex, age, mass, average of left and right forearm	Body condition index†	No. clonal blood isolates	PCR of blood samples on *rpoB*	Fur ectoparasites‡	PCR of ectoparasite samples on *rpoB*
Fecal								
Myotis daubentonii	2010 Jun 3	60° 26′ 41” N, 22° 03′ 15” E	2018, M, adult, 8.8 g, 38.5 mm§	0.229	ND	ND	ND	ND
Myotis daubentonii	2011 Jul 20	60° 21′ 31” N, 22° 13′ 26” E	2140, M, adult, 8.0 g, 38.1 mm	0.210	ND	ND	ND	ND
Myotis daubentonii	2011 Jul 18	60° 26′ 54” N, 21° 59′ 27” E	2758, M, juvenile, ND, 37.5 mm	ND	ND	ND	ND	ND
Myotis daubentonii	2011 Jul 19	60° 26′ 54” N, 21° 59′ 27” E	2768, M, juvenile, ND, 36.4 mm	ND	ND	ND	ND	ND
Myotis daubentonii	2011 Jul 24	60° 12′ 45” N, 21° 51′ 18” E	2771, F, adult, 7.8 g, 34.7 mm	0.225	ND	ND	ND	ND
Myotis daubentonii	2011 Jul 24	60° 12′ 45” N, 21° 51′ 18” E	2772, F, adult, 14.3 g, 40 mm	0.358	ND	ND	ND	ND
Myotis brandtii	2011 Jul 27	60° 26′ 54” N, 22° 06′ 29” E	2786, F, adult, 7.9 g, 33.9 mm	0.233	ND	ND	ND	ND
Eptesicus nilssonii	2011 Jul 31	60° 26′ 54” N, 22° 06′ 29” E	2788, M, adult, 9.7 g, 39.0 mm	0.249	ND	ND	ND	ND
Myotis brandtii	2011 Jul 31	60° 26′ 54” N, 22° 06′ 29” E	2791, F, juvenile, 7.9 g, 35.7 mm	0.221	ND	ND	ND	ND
Blood								
Eptesicus nilssonii	2012 Aug 6	60° 27′ 14” N, 22° 17′ 05”E	2369, F, adult, 10.8 g, 41.5 mm	0.260	–	–	–	–
Myotis mystacinus	2012 Aug 25	59° 55′ 34” N, 22° 24′ 51” E	1156, F, juvenile, 6.2 g, 34.9 mm	0.178	–	*rpoB*-4	–	–
Myotis brandtii	2012 Aug 25	59° 55′ 34” N, 22° 24′ 51” E	no band, M, juvenile ND, ND	ND	–	–	Siphonaptera (n = 1)	*rpoB*-4
Eptesicus nilssonii	2012 Aug 25	59° 55′ 34” N, 22° 24′ 51” E	1157, F, adult, 10.1 g, 38.1 mm	0.265	6 clones, all *rpoB*-1#	ND	Siphonaptera (n = 1)	*rpoB-1*
Eptesicus nilssonii	2012 Aug 25	59° 55′ 34” N, 22° 24′ 51” E	1158, F, adult, 9.6 g, 39.5 mm	0.243	–	–	–	–
Myotis brandtii	2012 Aug 25	59° 55′ 34” N, 22° 24′ 51” E	1159, M, adult, 6.3 g, 35.2 mm	0.179	–	–	–	–
Myotis daubentonii	2012 Aug 25	59° 55′ 34” N, 22° 24′ 51” E	1160, M, juvenile, 8.1 g, 37.1 mm	0.218	1 clone, *rpoB*-3	ND	*Penicillidia monoceros* (n = 1)	*rpoB-5*
							Nycteribia kolenatii (n = 3)	–
Myotis daubentonii	2012 Aug 25	59° 55′ 34” N, 22° 24′ 51” E	1161, F, juvenile, 8.2 g, 37.7 mm	0.218	–	–	*Penicillidia monoceros* (n = 1)	–
Myotis mystacinus	2012 Aug 25	59° 55′ 34” N, 22° 24′ 51” E	1162, F, adult, 6.8 g, 34.4 mm	0.198	–	–	–	–
Myotis mystacinus	2012 Aug 25	59° 55′ 34” N, 22° 24′ 51” E	1163, F, adult, 6.6 g, 35.4 mm	0.186	–	–	–	–
Myotis daubentonii	2012 Aug 27	60° 26′ 54” N, 21° 59′ 27” E	2569, M, juvenile, 7.7 g, 37.3 mm	0.206	–	–	*Penicillidia monoceros* (n = 1)	*rpoB-2*
Myotis daubentonii	2012 Aug 27	60° 26′ 54” N, 21° 59′ 27” E	2570, M, juvenile, 7.8 g, 37.4 mm	0.209	–	–	–	–
Myotis daubentonii	2012 Aug 27	60° 26′ 54” N, 21° 59′ 27” E	2571, M, juvenile, 7.7 g, 37.9 mm	0.203	–	–	–	–
Myotis daubentonii	2012 Sep 3	60° 26′ 54” N, 21° 59′ 27” E	2572, F, adult, 9.0 g, 36.7 mm	0.245	–	–	*Nycteribia kolenatii* (n = 2)	–
Myotis daubentonii	2012 Sep 3	60° 26′ 54” N, 21° 59′ 27” E	2573, M, juvenile, 7.9 g, 36.9 mm	0.214	2 clones, both *rpoB*-3	ND	–	–
Myotis daubentonii	2012 Sep 3	60° 26′ 54” N, 21° 59′ 27” E	2574, M, juvenile, 7.5 g, 36 mm	0.208	4 clones, all *rpoB*-2	ND	–	–
Myotis daubentonii	2012 Sep 3	60° 26′ 54” N, 21° 59′ 27” E	2575, M, juvenile, 7.4 g, 37.8 g	0.196	3 clones, all *rpoB*-3	ND	*Nycteribia kolenatii* (n = 1)	*rpoB-2*
Myotis daubentonii	2012 Sep 3	60° 26′ 54” N, 21° 59′ 27” E	2576, M, juvenile, 7.4 g, 36.8 mm	0.201	12 clones, all *rpoB*-3	ND	*Nycteribia kolenatii* (n = 1)	–

*M, male; F, female; ND, not determined; –, negative results.
†Mass divided with the average of the left and right forearm.
‡Visual identification to the order Siphonaptera during sampling. Species identification of the flies additionally based on mitochondrial cytochrome c oxidase subunit I barcode analysis at http://v3.boldsystems.org/.
§Individual of the metagenomic fecal sample.
#The detected *Bartonella* spp. *rpoB* allele 1 - 5.

Technical Appendix Table 2. Oligonucleotide primers used in this study

Oligo	Target genetic marker, oligo orientation	Sequence 5′→3′	Reference
Bartonella spp.			
fD1	16S rRNA gene, forward	AGAGTTTGATCCTGGCTCAG	(*1*)
rP2	16S rRNA gene, reverse	ACGGCTACCTTGTTACGACTT	(*1*)
Bart/16–23F	16S-23S rRNA intergenic spacer region (ISR), forward	TTGATAAGCGTGAGGTCGGAGG	(*2*)
Bart/16–23R	16S-23S rRNA intergenic spacer region (ISR), reverse	CAAAGCAGGTGCTCTCCCAG	(*2*)
prAPT0243	GltA gene, forward	GCCATGTCTGCTTTTTATCA	This study
BhCS.781p	GltA gene, forward	GGGGACCAGCTCATGGTGG	(*3*)
BhCS.1137n	GltA gene, reverse	AATGCAAAAAGAACAGTAAACA	(*3*)
prAPT0244	RpoB gene, forward	GATGTGCATCCTACGCATTATGG	(*4*)
prAPT0245	RpoB gene, reverse	AATGGTGCCTCAGCACGTATAAG	(*4*)
prAPT0257	FtsZ gene, forward	GCCTTCAAGGAGTTGATTTTGTTGTTGCCAAT	This study
prAPT0258	FtsZ gene, reverse	ACGACCCATTTCATGCATAACAGAAC	This study
ssrA-F	SsrA gene, forward	GCTATGGTAATAAATGGACAATGAAATAA	(*5*)
ssrA-R	SsrA gene, reverse	GCTTCTGTTGCCAGGTG	(*5*)
prPE23	VirB4 gene, forward	GGTTGCTTTATATTCTCACATC	(*6*)
prPE24	VirB4 gene, reverse	GAAGTTGCGCCCACCATG	(*6*)
Ectoparasites			
ZBJ-ArtF1c	Mitochondrial cytochrome c oxidase subunit I (COI)	AGATATTGGAACWTTATATTTTATTTTTGG	(*7*)
ZBJ-ArtR2c	Mitochondrial cytochrome c oxidase subunit I (COI)	WACTAATCAATTWCCAAATCCTCC	(*7*)

References

1. Weisburg WG, Barns SM, Pelletier DA, Lane DJ. 16S ribosomal DNA amplification for phylogenetic study. J Bacteriol. 1991;173:697–703. PubMed

2. García-Esteban C, Gil H, Rodríguez-Vargas M, Gerrikagoitia X, Barandika J, Escudero R, et al. Molecular method for *Bartonella* species identification in clinical and environmental samples. J Clin Microbiol. 2008;46:776–9. PubMed http://dx.doi.org/10.1128/JCM.01720-07

3. Norman AF, Regnery R, Jameson P, Greene C, Krause DC. Differentiation of *Bartonella*-like isolates at the species level by PCR-restriction fragment length polymorphism in the citrate synthase gene. J Clin Microbiol. 1995;33:1797–803. PubMed

4. Oksi J, Rantala S, Kilpinen S, Silvennoinen R, Vornanen M, Veikkolainen V, et al. Cat scratch disease caused by *Bartonella grahamii* in an immunocompromised patient. J Clin Microbiol. 2013;51:2781–4. PubMed http://dx.doi.org/10.1128/JCM.00910-13

5. Diaz MH, Bai Y, Malania L, Winchell JM, Kosoy MY. Development of a novel genus-specific real-time PCR assay for detection and differentiation of *Bartonella* species and genotypes. J Clin Microbiol. 2012;50:1645–9. PubMed http://dx.doi.org/10.1128/JCM.06621-11

6. Saenz HL, Engel P, Stoeckli MC, Lanz C, Raddatz G, Vayssier-Taussat M, et al. Genomic analysis of *Bartonella* identifies type IV secretion systems as host adaptability factors. Nat Genet. 2007;39:1469–76. PubMed http://dx.doi.org/10.1038/ng.2007.38

7. Zeale MR, Butlin RK, Barker GL, Lees DC, Jones G. Taxon-specific PCR for DNA barcoding arthropod prey in bat faeces. Mol Ecol Resour. 2011;11:236–44. PubMed http://dx.doi.org/10.1111/j.1755-0998.2010.02920.x

Technical Appendix Table 3. Results of the BLASTN homology searches performed in January 2013

Bat strain	Marker (sequenced length)*	GenBank accession no.	Closely related *Bartonella* spp. or *Candidatus*-status *Bartonella* spp., percentage of similarity (bat strain/reference strain), GenBank accession no. of the reference strain		
			Closest	Second closest	Third closest
1157/3	16S rRNA (485 bp)	KF003116	Nondisc†, 100% (485/485)	Nondisc, 99.8% (484/485)	Nondisc, 99.6% (483/485)
	ITS (259 bp)	KF003117	*Candidatus* B. mayotimonensis, 85.7% (239/279) FJ376735	None	None
	rpoB (406 bp)	KF003118	*Candidatus* B. mayotimonensis, 95.8% (387/404) FJ376736	*B. alsatica*, 95.3% (385/404) AF165987	*B. vinsonii* subsp. arupensis, 95.1% (385/405) AY166582
	gltA (595 bp)	KF003115	*B. vinsonii* subsp. arupensis, 92.9% (553/595) AF214557	*Candidatus* B. mayotimonensis, 92.8% (552/595) FJ376732	*B. taylorii*, 92.6% (551/595) Z70013
	ftsZ (511 bp)	KF003121	*Candidatus* B. mayotimonensis, 96% (267/278) FJ376734	*B. washoensis*, 93.2% (476/511) AB292598	Nondisc, 92.6% (472/511)
	ssrA (254 bp)	KF003119	*B. washoensis*, 97.2% (247/254) JN029786	*B. grahamii*, 95.7% (243/254) JN029795	Nondisc, 95.3% (242/254)
1160/1	16S rRNA (485 bp)	KF003123	*B. japonica*, 100% (485/485) AB440632	Nondisc, 99.8% (484/485)	Nondisc, 99.6% (483/485)
	ITS (265 bp)	KF003124	*Candidatus* B. mayotimonensis, 83.3% (235/282) FJ376735	None	None
	rpoB (406 bp)	KF003125	*Candidatus* B. mayotimonensis, 97.0% (393/405) FJ376736	*B. vinsonii* subsp. arupensis, 93.6% (380/406) AY166582	*B. alsatica*, 93.6% (379/405) AF165987
	gltA (595 bp)	KF003122	*Candidatus* B. mayotimonensis, 91.4%(544/595) FJ376732	*B. vinsonii* subsp. arupensis, 90.8% (540/595) AF214557	Nondisc, 90.6% (539/595)
	ftsZ (511 bp)	KF003128	*Candidatus* B. mayotimonensis, 95.3% (265/278) FJ376734	Nondisc, 91.2% (466/511)	*B. phoceensis*, 91.0% (465/511) AY515135
	ssrA (253 bp)	KF003126	*B. washoensis*, 96.4% (244/253) JN029786	Nondisc, 96.0% (243/253)	*B. grahamii*, 95.3% (241/253) JN029795
2574/1	16S rRNA (485 bp)	KF003130	Nondisc, 100% (485/485)	Nondisc, 99.8% (484/485)	Nondisc, 99.6% (483/485)
	ITS (163 bp)	KF003131	None	None	None
	rpoB (406 bp)	KF003132	*B. quintana*, 91.9% (372/405) AF165994	Nondisc, 91.1% (370/406)	Nondisc, 90.9% (369/406)
	gltA (595 bp)	KF003129	*B. koehlerae*, 91.8% (546/595) AF176091	*B. henselae*, 91.3% (543/595) CAF27442	*B. quintana*, 90.4% (538/595) Z70014
	ftsZ (511 bp)	KF003135	*B. vinsonii* subsp. vinsonii, 88.5% (452/511) AF467757	Nondisc, 88.3% (451/511)	*B. grahamii*, 87.7% (448/511) AF467753
	ssrA (253 bp)	KF003133	*B. vinsonii* subsp. arupensis, 94.9% (240/253) JN029783	*B. vinsonii* subsp. vinsonii, 94.5% (239/253) JN029777	Nondisc, 93.3% (236/253)

*Type strain *ssrA* sequences are not available for all species and *Candidatus* B. mayotimonensis.
†Nondisc, a nondiscriminatory marker (≥2 *Bartonella* species or *Candidatus*-status *Bartonella* species have the same sequence similarity with the bat strain.

Technical Appendix Table 4. Pairwise genetic distance values of the concatenated *rpoB*, *gltA*, 16S rRNA and *ftsZ* sequence fragments. Lowest genetic distance values of the bat strains compared with the *Brucella abortus* outgroup strain 9–941, the *Bartonella* type strains (Technical Appendix Table 5), and the *Candidatus* B. mayotimonensis patient strain are underlined.

		1	2	3	4	5	6	7	8	9	10	11	12	13	14	15	16	17	**18**	**19**	**20**	**21**	**22**	**23**	**24**	**25**	**26**	**27**	**28**	**29**	**30**	**31**	**32**	**33**	**34**	**35**	**36**	
1	*B. alsatica*																																					**1**
2	*B. australis*	0.109																																				**2**
3	*B. bacilliformis*	0.102	0.118																																			**3**
4	*B. birtlesii*	0.065	0.098	0.106																																		**4**
5	*B. bovis*	0.096	0.104	0.095	0.084																																	**5**
6	*B. capreoli*	0.090	0.097	0.097	0.067	0.045																																**6**
7	*B. chomelii*	0.099	0.106	0.104	0.079	0.045	0.021																															**7**
8	*B. clarridgeiae*	0.096	0.106	0.096	0.097	0.096	0.092	0.100																														**8**
9	*B. coopersplainsensis*	0.070	0.113	0.112	0.086	0.112	0.101	0.111	0.107																													**9**
10	*B. doshiae*	0.090	0.107	0.109	0.084	0.097	0.084	0.096	0.099	0.100																												**10**
11	*B. elizabethae*	0.084	0.127	0.110	0.092	0.099	0.099	0.103	0.102	0.097	0.097																											**11**
12	*B. grahamii*	0.076	0.118	0.109	0.084	0.094	0.087	0.095	0.098	0.090	0.088	0.042																										**12**
13	*B. henselae*	0.070	0.106	0.102	0.077	0.084	0.086	0.093	0.099	0.081	0.080	0.091	0.080																									**13**
14	*B. japonica*	0.074	0.111	0.109	0.085	0.104	0.095	0.105	0.102	0.027	0.097	0.099	0.088	0.080																								**14**
15	*B. koehlerae*	0.070	0.107	0.099	0.081	0.083	0.086	0.093	0.094	0.084	0.080	0.083	0.078	0.031	0.081																							**15**
16	*B. melophagi*	0.095	0.100	0.095	0.074	0.040	0.017	0.014	0.096	0.106	0.092	0.099	0.090	0.087	0.099	0.086																						**16**
17	*B. phoceensis*	0.064	0.107	0.096	0.082	0.092	0.084	0.092	0.095	0.073	0.092	0.080	0.075	0.076	0.074	0.067	0.086																					**17**
18	*B. queenslandensis*	0.082	0.119	0.105	0.089	0.101	0.092	0.101	0.099	0.098	0.091	0.040	0.039	0.085	0.093	0.082	0.095	0.089																				**18**
19	*B. quintana*	0.077	0.109	0.111	0.087	0.095	0.090	0.102	0.093	0.091	0.088	0.094	0.085	0.056	0.087	0.059	0.096	0.078	0.088																			**19**
20	*B. rattaustraliani*	0.079	0.113	0.115	0.083	0.110	0.106	0.108	0.108	0.054	0.095	0.097	0.091	0.082	0.054	0.089	0.104	0.082	0.101	0.093																		**20**
21	*B. rattimassiliensis*	0.083	0.130	0.117	0.091	0.099	0.093	0.103	0.111	0.099	0.098	0.056	0.045	0.088	0.097	0.090	0.099	0.087	0.053	0.093	0.101																	**21**
22	*B. rochalimae*	0.094	0.111	0.095	0.099	0.089	0.086	0.094	0.045	0.107	0.101	0.097	0.096	0.088	0.101	0.095	0.092	0.097	0.094	0.093	0.107	0.104																**22**
23	*B. schoenbuchensis*	0.100	0.103	0.099	0.079	0.039	0.016	0.011	0.098	0.111	0.093	0.102	0.095	0.091	0.104	0.090	0.012	0.087	0.098	0.098	0.110	0.100	0.091															**23**
24	*B. silvatica*	0.082	0.125	0.121	0.094	0.099	0.093	0.101	0.104	0.089	0.102	0.091	0.087	0.090	0.088	0.096	0.098	0.078	0.098	0.090	0.092	0.093	0.103	0.097														**24**
25	*B. tamiae*	0.153	0.160	0.168	0.158	0.163	0.155	0.158	0.166	0.156	0.155	0.159	0.162	0.155	0.154	0.155	0.159	0.143	0.156	0.150	0.161	0.161	0.158	0.156	0.157													**25**
26	*B. taylorii*	0.061	0.117	0.099	0.068	0.092	0.079	0.090	0.095	0.078	0.082	0.088	0.082	0.071	0.077	0.070	0.085	0.065	0.087	0.069	0.081	0.090	0.095	0.087	0.081	0.152												**26**
27	*B. tribocorum*	0.072	0.115	0.102	0.078	0.096	0.090	0.102	0.095	0.088	0.088	0.035	0.033	0.078	0.086	0.076	0.098	0.079	0.030	0.081	0.092	0.053	0.088	0.098	0.093	0.157	0.078											**27**
28	*B. vinsonii* subsp. arupensis	0.054	0.110	0.100	0.068	0.094	0.087	0.093	0.090	0.068	0.078	0.082	0.075	0.068	0.070	0.068	0.089	0.062	0.080	0.064	0.074	0.084	0.092	0.092	0.080	0.150	0.054	0.075										**28**
29	*B. vinsonii* subsp. berkhoffii	0.052	0.106	0.100	0.068	0.096	0.090	0.094	0.092	0.070	0.083	0.081	0.076	0.068	0.072	0.069	0.093	0.065	0.078	0.069	0.072	0.084	0.095	0.097	0.081	0.151	0.059	0.072	0.035									**29**
30	*B. vinsonii* subsp. vinsonii	0.048	0.107	0.099	0.064	0.090	0.081	0.088	0.095	0.072	0.076	0.083	0.074	0.068	0.072	0.069	0.083	0.064	0.078	0.068	0.076	0.082	0.093	0.086	0.082	0.155	0.050	0.072	0.030	0.028								**30**
31	*B. washoensis*	0.073	0.107	0.106	0.077	0.090	0.085	0.095	0.094	0.081	0.077	0.080	0.076	0.053	0.081	0.054	0.088	0.071	0.081	0.055	0.084	0.084	0.090	0.095	0.088	0.151	0.067	0.075	0.057	0.061	0.061							**31**
32	*Candidatus* B. mayotimonensis	0.057	0.109	0.100	0.075	0.100	0.093	0.100	0.099	0.076	0.090	0.086	0.086	0.076	0.076	0.073	0.096	0.067	0.086	0.075	0.076	0.090	0.103	0.099	0.085	0.159	0.059	0.080	0.055	0.050	0.056	0.072						**32**
33	Bat strain 1157/3	0.050	0.109	0.103	0.061	0.086	0.081	0.091	0.089	0.068	0.084	0.078	0.075	0.063	0.065	0.065	0.083	0.064	0.082	0.068	0.064	0.080	0.093	0.089	0.078	0.161	0.051	0.070	0.044	0.048	0.046	0.057	**0.040**					**33**
34	Bat strain 1160/1	0.061	0.103	0.103	0.073	0.097	0.089	0.099	0.102	0.076	0.085	0.084	0.079	0.076	0.070	0.076	0.092	0.066	0.083	0.081	0.071	0.087	0.097	0.096	0.085	0.156	0.057	0.075	0.056	0.056	0.058	0.070	**0.038**	0.030				**34**
35	Bat strain 2574/1	0.089	0.099	0.106	0.084	0.086	0.079	0.094	0.097	0.102	0.079	0.100	0.091	0.077	0.099	0.073	0.087	0.087	0.095	0.079	0.097	0.098	0.091	0.088	0.107	0.163	0.089	0.086	0.083	0.080	0.080	**0.070**	0.088	0.081	0.087			**35**
36	*Brucella abortus* 9–941	0.243	0.246	0.245	0.255	0.260	0.247	0.256	0.252	0.256	0.254	0.236	0.235	0.247	0.245	0.249	0.249	0.243	0.236	0.237	0.251	0.239	0.245	0.254	0.250	0.231	0.248	0.244	0.244	0.236	0.245	0.239	0.243	0.240	0.252	0.263		**36**

Technical Appendix Table 5. *Bartonella* spp. type strain sequences used in the multilocus sequence and phylogenetic analyses

Species	Type strain, isolated from	Reference	GenBank accession no.			
			gltA	*rpoB*	16S rRNA	*ftsZ*
B. alsatica	IBS 382, rabbit (*Oryctolagus cuniculus)*	(*1*)	AF204273	AF165987	AJ002139	AF467763
B. australis	Aust/NH1, kangaroo (*Macropus giganteus*)	(*2*)	NC_020300	NC_020300	DQ538394	NC_020300
B. bacilliformis	KC583, unknown origin	(*3*)	YP_988907	AF165988	NR_044743	AB292602
B. birtlesii	IBS 325, mouse (*Apodemus* spp.)	(*4*)	AF204272	AB196425	NR_025051	AF467762
B. bovis	91–4, domestic cow	(*5*)	AF293394	AY166581	NR_025121	AGWA01000007
B. capreoli	IBS 193, roe deer (*Capreolus capreolus*)	(*5*)	AF293392	AB290188	NR_025120	AB290192
B. chomelii	A828, domestic cow	(*6*)	AY254308	AB290189	NR_025736	AB290193
B. clarridgeiae	Houston-2, cat	(*7*)	U84386	AF165990	AB292603	AF141018
B. coopersplainsensis	AUST/NH20, rat (*Rattus leucopus*)	(*8*)	EU111803	EU111792	EU111759	EU111781
B. doshiae	R18, field vole (*Migrotus agrestis*)	(*9*)	Z70017	AF165991	NR_029368	AF467754
B. elizabethae	F9251, human	(*10*)	Z70009	AF165992	NR_025889	AF467760
B. grahamii	V2, bank vole (*Myodes glareolus*)	(*9*)	Z70016	AF165993	NR_029366	AF467753
B. henselae	Houston-1, human	(*11*)	CAF27442	AF171070	NC_005956	AF061746
B. japonica	Fuji 18–1, mouse (*Apodemus argenteus*)	(*12*)	AB242289	AB242288	AB440632	AB440633
B. koehlerae	C-29, cat	(*13*)	AF176091	AY166580	NR_024932	AF467755
B. melophagi	K-2C, sheep ked	(*14*)	AY724768	EF605288	AIMA01000004	EF605286
B. phoceensis	16120, rat (*Rattus norvegicus*)	(*15*)	AY515126	AY515132	AY515119	AY515135
B. queenslandensis	Aust/NH12, rat (*Melomys* sp.)	(*8*)	EU111798	EU111787	EU111754	EU111776
B. quintana	Fuller, human	(*16*)	Z70014	AF165994	NR_044748	AB292605
B. rattimassiliensis	15908, rat (*Rattus norvegicus*)	(*15*)	AY515124	AY515130	AY515120	AY515133
B. rochalimae	ATCC BAA-1498, human	(*17*)	DQ683195	DQ683198	FN645466	FN645461
B. schoenbuchensis	R1, roe deer (*Capreolus capreolus*)	(*18*)	AJ278183	AY167409	AJ278187	AF467765
B. silvatica	Fuji 23–1, mouse (*Apodemus speciosus*)	(*12*)	AB242287	AB242292	AB440636	AB440637
B. tamiae	Th239, human	(*19*)	DQ395177	EF091855	AIMB01000009	DQ395178
B. taylorii	M6, mouse (*Apodemus* spp.)	(*9*)	Z70013	AF165995	NR_029367	AF467756
B. tribocorum	IBS 506, rat (*Rattus norvegicus*)	(*20*)	AJ005494	AF165996	AM260525	AF467759
B. vinsonii subsp. arupensis	OK-94–513, human	(*21*)	AF214557	AY166582	AF214558	AF467758
B. vinsonii subsp. berkhofii	93-CO1, dog	(*22*)	U28075	AF165989	L35052	AF467764
B. vinsonii subsp. vinsonii	Baker, vole (species unknown)	(*23*)	Z70015	AF165997	NR_037056	AF467757
B. washoensis	Sb944nv, ground squirrel (*Spermophilus beecheyi*)	(*24*)	AF470616	AB292596	AB292597	AB292598

References

1. Heller R, Kubina M, Mariet P, Riegel P, Delacour G, Dehio C, et al. *Bartonella alsatica* sp. nov., a new *Bartonella* species isolated from the blood of wild rabbits. Int J Syst Bacteriol. 1999;49:283–8. PubMed http://dx.doi.org/10.1099/00207713-49-1-283

2. Fournier PE, Taylor C, Rolain JM, Barrassi L, Smith G, Raoult D. *Bartonella australis* sp. nov. from kangaroos, Australia. Emerg Infect Dis. 2007;13:1961–2. PubMed http://dx.doi.org/10.3201/eid1312.060559

3. Brenner DJ, O'Connor SP, Hollis DG, Weaver RE, Steigerwalt AG. Molecular characterization and proposal of a neotype strain for *Bartonella bacilliformis*. J Clin Microbiol. 1991;29:1299–302. PubMed

4. Bermond D, Heller R, Barrat F, Delacour G, Dehio C, Alliot A, et al. *Bartonella birtlesii* sp. nov., isolated from small mammals (*Apodemus* spp.). Int J Syst Evol Microbiol. 2000;50:1973–9. PubMed http://dx.doi.org/10.1099/00207713-50-6-1973

5. Bermond D, Boulouis HJ, Heller R, Van Laere G, Monteil H, Chomel BB, et al. *Bartonella bovis* Bermond et al. sp. nov. and *Bartonella capreoli* sp. nov., isolated from European ruminants. Int J Syst Evol Microbiol. 2002;52:383–90. PubMed

6. Maillard R, Riegel P, Barrat F, Bouillin C, Thibault D, Gandoin C, et al. *Bartonella chomelii* sp. nov., isolated from French domestic cattle (*Bos taurus*). Int J Syst Evol Microbiol. 2004;54:215–20. PubMed http://dx.doi.org/10.1099/ijs.0.02770-0

7. Kordick DL, Hilyard EJ, Hadfield TL, Wilson KH, Steigerwalt AG, Brenner DJ, et al. *Bartonella clarridgeiae*, a newly recognized zoonotic pathogen causing inoculation papules, fever, and lymphadenopathy (cat scratch disease). J Clin Microbiol. 1997;35:1813–8. PubMed

8. Gundi VA, Taylor C, Raoult D, La Scola B. *Bartonella rattaustraliani* sp. nov., *Bartonella queenslandensis* sp. nov. and *Bartonella coopersplainsensis* sp. nov., identified in Australian rats. Int J Syst Evol Microbiol. 2009;59:2956–61. PubMed http://dx.doi.org/10.1099/ijs.0.002865-0

9. Birtles RJ, Harrison TG, Saunders NA, Molyneux DH. Proposals to unify the genera *Grahamella* and *Bartonella*, with descriptions of *Bartonella talpae* comb. nov., *Bartonella peromysci* comb. nov., and three new species, *Bartonella grahamii* sp. nov., *Bartonella taylorii* sp. nov., and *Bartonella doshiae* sp. nov. Int J Syst Bacteriol. 1995;45:1–8. PubMed http://dx.doi.org/10.1099/00207713-45-1-1

10. Daly JS, Worthington MG, Brenner DJ, Moss CW, Hollis DG, Weyant RS, et al. *Rochalimaea elizabethae* sp. nov. isolated from a patient with endocarditis. J Clin Microbiol. 1993;31:872–81. PubMed

11. Regnery RL, Anderson BE, Clarridge JE, Rodriguez-Barradas MC, Jones DC, Carr JH. Characterization of a novel *Rochalimaea* species, *R. henselae* sp. nov., isolated from blood of a febrile, human immunodeficiency virus–positive patient. J Clin Microbiol. 1992;30:265–74. PubMed

12. Inoue K, Kabeya H, Shiratori H, Ueda K, Kosoy MY, Chomel BB, et al. *Bartonella japonica* sp. nov. and *Bartonella silvatica* sp. nov., isolated from Apodemus mice. Int J Syst Evol Microbiol. 2010;60:759–63. PubMed http://dx.doi.org/10.1099/ijs.0.011528-0

13. Droz S, Chi B, Horn E, Steigerwalt AG, Whitney AM, Brenner DJ. *Bartonella koehlerae* sp. nov., isolated from cats. J Clin Microbiol. 1999;37:1117–22. PubMed

14. Maggi RG, Kosoy M, Mintzer M, Breitschwerdt EB. Isolation of Candidatus *Bartonella melophagi* from human blood. Emerg Infect Dis. 2009;15:66–8. PubMed http://dx.doi.org/10.3201/eid1501.081080

15. Gundi VA, Davoust B, Khamis A, Boni M, Raoult D, La Scola B. Isolation of *Bartonella rattimassiliensis* sp. nov. and *Bartonella phoceensis* sp. nov. from European Rattus norvegicus. J Clin Microbiol. 2004;42:3816–8. PubMed http://dx.doi.org/10.1128/JCM.42.8.3816-3818.2004

16. Brenner DJ, O'Connor SP, Winkler HH, Steigerwalt AG. Proposals to unify the genera *Bartonella* and *Rochalimaea*, with descriptions of *Bartonella quintana* comb. nov., *Bartonella vinsonii* comb. nov., *Bartonella henselae* comb. nov., and *Bartonella elizabethae* comb. nov., and to remove the family Bartonellaceae from the order Rickettsiales. Int J Syst Bacteriol. 1993;43:777–86. PubMed http://dx.doi.org/10.1099/00207713-43-4-777

17. Eremeeva ME, Gerns HL, Lydy SL, Goo JS, Ryan ET, Mathew SS, et al. Bacteremia, fever, and splenomegaly caused by a newly recognized *Bartonella* species. N Engl J Med. 2007;356:2381–7. PubMed http://dx.doi.org/10.1056/NEJMoa065987

18. Dehio C, Lanz C, Pohl R, Behrens P, Bermond D, Piémont Y, et al. *Bartonella schoenbuchii* sp. nov., isolated from the blood of wild roe deer. Int J Syst Evol Microbiol. 2001;51:1557–65. PubMed

19. Kosoy M, Morway C, Sheff KW, Bai Y, Colborn J, Chalcraft L, et al. *Bartonella tamiae* sp. nov., a newly recognized pathogen isolated from three human patients from Thailand. J Clin Microbiol. 2008;46:772–5. PubMed http://dx.doi.org/10.1128/JCM.02120-07

20. Heller R, Riegel P, Hansmann Y, Delacour G, Bermond D, Dehio C, et al. *Bartonella tribocorum* sp. nov., a new *Bartonella* species isolated from the blood of wild rats. Int J Syst Bacteriol. 1998;48:1333–9. PubMed http://dx.doi.org/10.1099/00207713-48-4-1333

21. Welch DF, Carroll KC, Hofmeister EK, Persing DH, Robison DA, Steigerwalt AG, et al. Isolation of a new subspecies, *Bartonella vinsonii* subsp. arupensis, from a cattle rancher: identity with isolates found in conjunction with *Borrelia burgdorferi* and *Babesia microti* among naturally infected mice. J Clin Microbiol. 1999;37:2598–601. PubMed

22. Breitschwerdt EB, Kordick DL, Malarkey DE, Keene B, Hadfield TL, Wilson K. Endocarditis in a dog due to infection with a novel *Bartonella* subspecies. J Clin Microbiol. 1995;33:154–60. PubMed

23. Baker JA. A Rickettsial infection in Canadian voles. J Exp Med. 1946;84:37–50. http://dx.doi.org/10.1084/jem.84.1.37

24. Kosoy M, Murray M, Gilmore RD, Bai Y, Gage KL. *Bartonella* strains from ground squirrels are identical to *Bartonella washoensis* isolated from a human patient. J Clin Microbiol. 2003;41:645–50. PubMed http://dx.doi.org/10.1128/JCM.41.2.645-650.2003

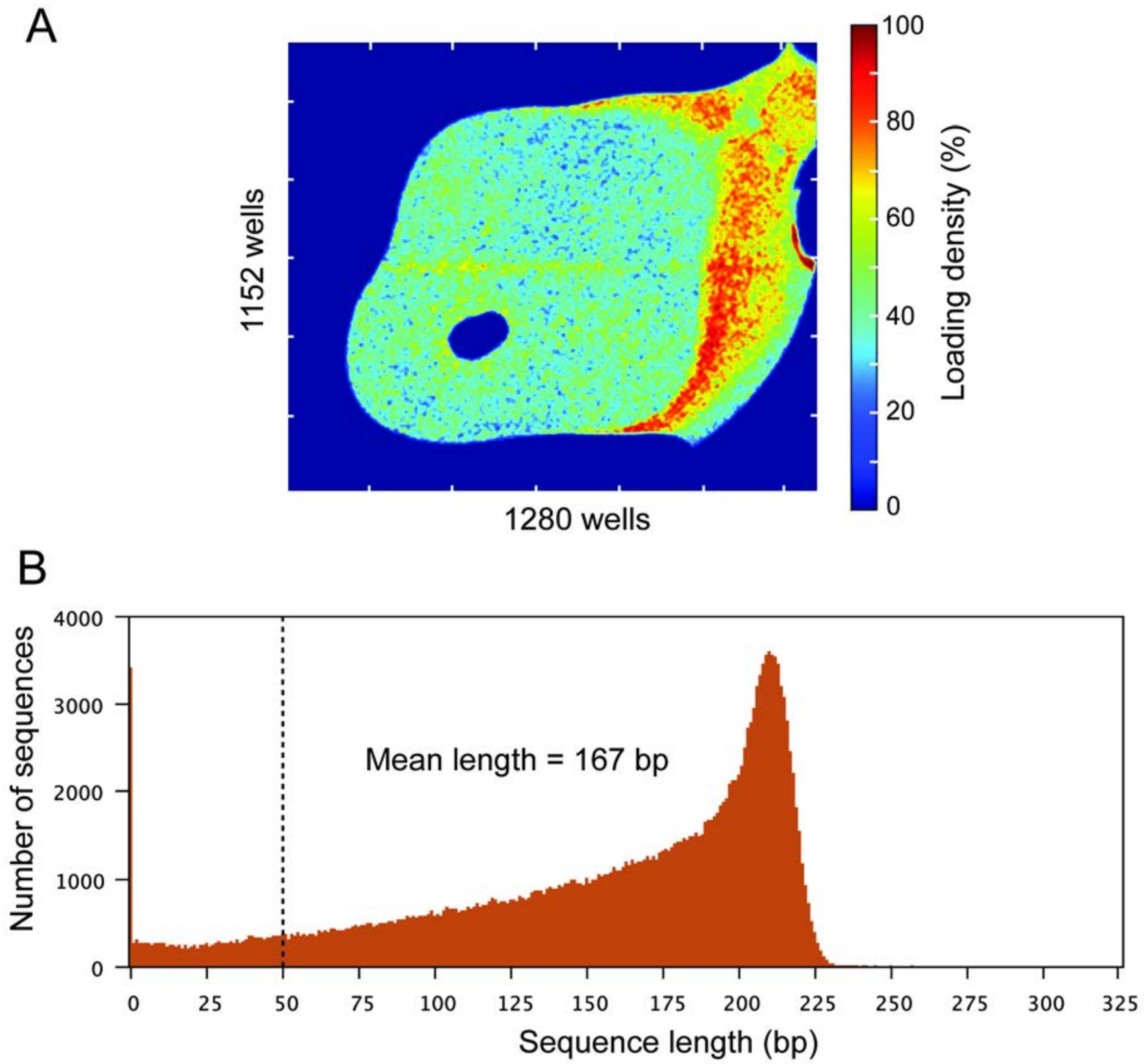

Technical Appendix Figure 1. Performance of the Ion Torrent Personal Genome Machine. A) Loading density of the chip (average 31%). Twenty-five percent of the loaded beads were polyclonal and were distracted from further analysis together with beads that gave low quality reads (18% of the loaded beads). Approximately 200,000 good quality sequences were obtained with 58% of the loaded beads. B) Read length histogram of the bat fecal metagenome. Sequences <50 bp (dashed line) were not used in the BLASTN/GenBank homology search-based assignments.

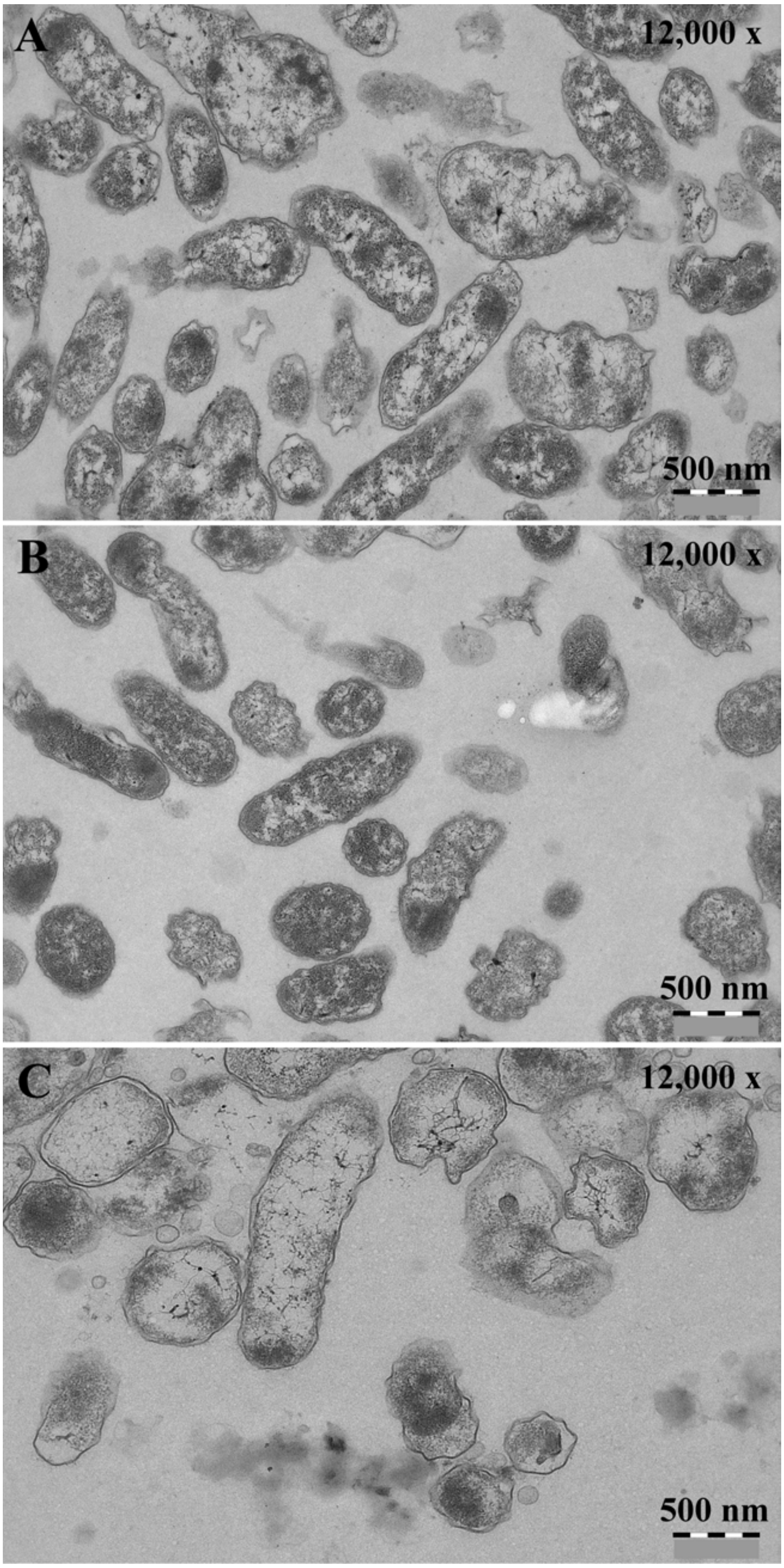

Technical Appendix Figure 2. Transmission electron micrographs of the bat *Bartonella* isolates. *B. mayotimonensis* strain 1157/3 (A), *B. mayotimonensis* strain 1160/1T (B) and *B. naantaliensis* sp. nov. strain 2574/1T (C). Original magnification × 12,000. Scale bars = 500 nm.
